# Oxygen‐Depleted Calixarenes as Ligands for Molecular Models of Galactose Oxidase

**DOI:** 10.1002/chem.201903820

**Published:** 2019-09-19

**Authors:** Matthias Keck, Santina Hoof, Christian Herwig, Arkadi Vigalok, Christian Limberg

**Affiliations:** ^1^ Institut für Chemie Humboldt-Universität zu Berlin Brook-Taylor-Strasse 2 12489 Berlin Germany; ^2^ School of Chemistry Tel Aviv University 1 Ramat Aviv 69978 Tel Aviv Israel

**Keywords:** alcohol oxidation, biomimetic chemistry, calixarenes, copper, nickel

## Abstract

A calix[4]arene ligand, in which two of the phenol functions are replaced by pyrazole units has been employed to mimic the His_2_–Tyr_2_ (His: histidine, Tyr: tyrosine) ligand sphere within the active site of the galactose oxidase (GO). The calixarene backbone forces the corresponding copper(II) complex into a see‐saw‐type structure, which is hitherto unprecedented in GO modelling chemistry. It undergoes a one‐electron oxidation that is centered at the phenolate donor leading to a copper‐coordinated phenoxyl radical like in the GO. Accordingly, the complex was tested as a functional model and indeed proved capable of oxidizing benzyl alcohol to the respective aldehyde using two phenoxyl‐radical equivalents as oxidants. Finally, the results show that the calixarene platform can be utilized to arrange donor functions to biomimetic binding pockets that allow for the creation of novel types of model compounds.

## Graphical abstract

**Calixarenes go biomimetic**: Replacement of two phenol functions by pyrazoles allows for the synthesis of late‐transition‐metal complexes, which mimic the galactose oxidase structurally and functionally.

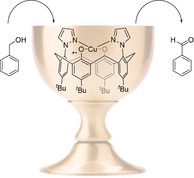

Macrocyclic polyphenols—coined calixarenes by C. D. Gutsche—have been known for almost 80 years now.^1^ Their high‐yield preparation from cheap commercially available starting materials and easy post modifications have made them well‐established and often utilized macromolecules in diverse fields of chemical research and applications.^2^ Calixarenes were also employed as ligands in transition‐metal chemistry, for instance, as a mimic of oxidic surfaces and hence for the modelling of the active sites of heterogeneous catalysts.^3^ However, given that they provide a coordination platform that contains exclusively hard oxygen donors, they have hardly been used in biomimetic studies. There are no enzymes with such binding pockets composed for obvious reasons: hard metal centers in high oxidation states (e.g. Fe^III^ or Fe^IV^) mainly occur in reactive intermediates as part of catalytic cycles and in the course of turnover these are reduced to softer, low‐oxidation‐state metal centers (e.g. Fe^II^), which prefer soft ligands. Metal‐binding sites of metalloenzymes have to balance these two different demands and therefore often feature mixed ligand spheres, which have to be mimicked also in models. Hence, it is not surprising that calixarenes, so far, have rarely been employed in bioinorganic chemistry. Only modified forms in which the phenol units are functionalized by pendant N‐donors were investigated, for instance, in biomimetic copper or zinc chemistry.^4^ From the structural point of view, the introduction of the pendant groups makes the metal complex more flexible. Even though not necessarily detrimental to the desired activity, this flexibility eliminates one attractive feature of the calixarene platform, namely its potential to direct donors to a well‐defined binding pocket, which renders calixarenes appealing as three‐dimensional analogues of rigid multidentate systems, such as salens or porphyrins.

We were interested in exploiting calixarene‐shaped binding sites also in bioinorganic investigations, which required the introduction of soft donor atoms directly in place of one or more oxygen atoms of the calixarene lower rim. Recently, some of us have developed the oxygen‐depleted calixarene bispyrazolyl‐*tert*‐butyl‐calix[4]arene ([H_2_(bpzCal)], Figure 1) featuring two pyrazole moieties beside two phenolic donors,^5^ which is reminiscent of a His_2_–Tyr_2_ coordination sphere found in certain enzymes. One of those is the galactose oxidase (GO) and hence we decided to test the potential of the [bpzCal]^2−^ ligand to construct molecular models of the GO.

**Figure 1:**
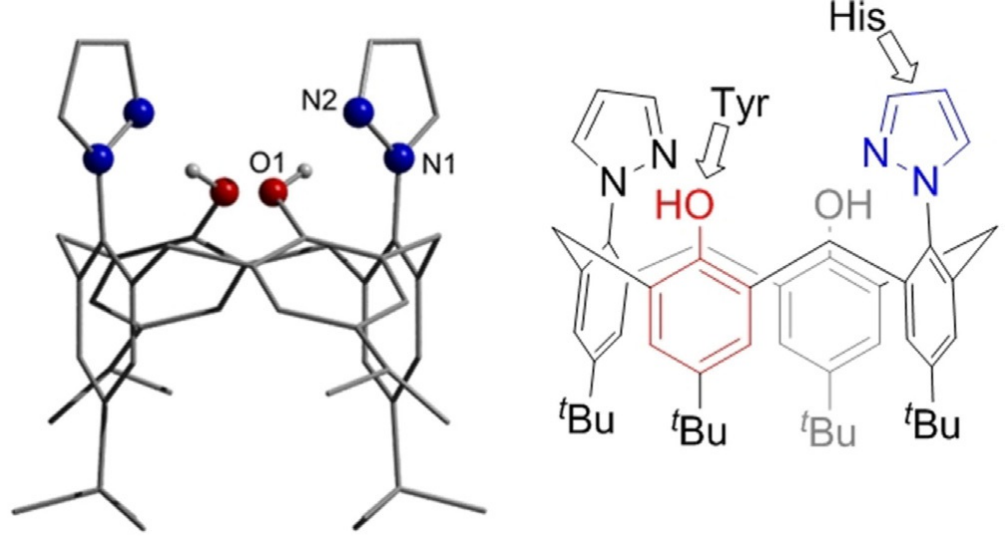
Left: Molecular structure of [H_2_(bpzCal)].^5^ Right: Similarities of the ligand's donors with natural amino acid side chains.

The GO catalyzes the oxidation of primary alcohols to the corresponding aldehydes. In the resting state it contains a copper(II) ion coordinated by two tyrosine and two histidine amino acid residues (Figure 2).^6^ Previous attempts to mimic this coordination environment often used salen ligands, which, however, forces the central ion into an almost square‐planar coordination environment. This rigidity has proven to impede changes in oxidation state, because the Franc–Condon barrier is high in energy as evidenced by model complexes that were oxidized most easily when a high degree of distortion at the metal center was given.^7^ In contrast to salens, calixarenes provide a more variable coordination site, which, however, is still well‐defined and pre‐organized for metal‐ion complexation.

**Figure 2:**
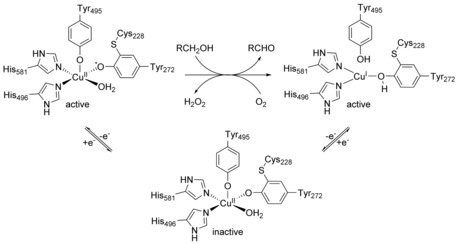
Catalytic cycle of GO and its different redox states.

Consequently, the synthesis of a copper(II) complex of [H_2_(bpzCal)] was pursued, as well as corresponding nickel and zinc complexes for comparison. Deprotonation of the ligand precursor, followed by salt metathesis, only led to incomplete reactions and salt contamination resulting in purification issues. Hence, another strategy had to be chosen. The use of basic metal precursors has already proven a suitable approach to obtain pure calixarene complexes in previous metalation reactions.^8^ Therefore, [Ni(NPh_2_)_2_]_2_ 9 and Cu(dmap)_2_ 10 (dmap: 1‐imethylamino‐2‐propanolate) were chosen as metalation reagents resulting in high yields of complexes [Ni(bpzCal)] and [Cu(bpzCal)] (Scheme 1), which were isolated as intensely colored analytically pure solids.

**Scheme 1:**
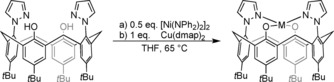
Syntheses of a) [Ni(bpzCal)] (M=Ni) and b) [Cu(bpzCal)] (M=Cu).

X‐ray structure analyses of single crystals that were grown by slow evaporation of the volatiles from THF solutions revealed almost identical coordination spheres for the metal centers in the two complexes, composed of two phenolate and two pyrazole donors (Figure 3 and Figure S14 in the Supporting Information).

**Figure 3:**
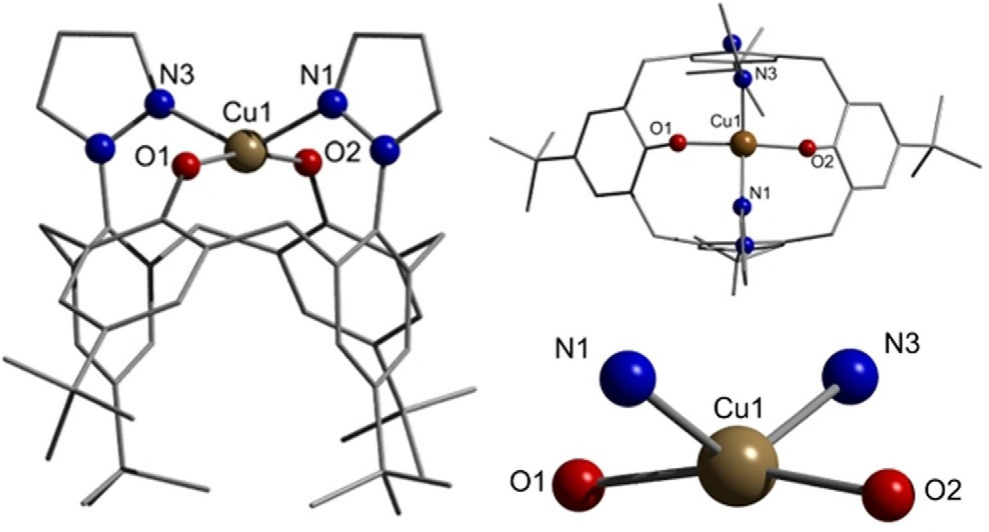
Molecular structure of [Cu(bpzCal)]. Selected bond lengths [Å] and angles [°]: Cu1−O1 1.896(3), Cu1−O2 1.856(3), Cu1−N1 2.035(4), Cu1−N3 2.018(4); N1‐Cu1‐N3 130.74(15), O1‐Cu1‐O2 165.68(14). Hydrogen atoms and cocrystallized solvent molecules are omitted for clarity.

In both cases, there are two crystallographically independent molecules in the unit cell, the metrical parameters of which are almost identical. Regarding the *τ*_δ_ value, the coordination geometry of the metal ions can be described best as intermediate between tetrahedral and square‐planar, commonly called the seesaw coordination.^11^ To the best of our knowledge, these two complexes are the first examples of structurally characterized mononuclear nickel and copper calixarene complexes in which phenolic oxygen donors directly coordinate the metal and therefore interact without any spacers.

Both compounds are in high‐spin configurations with magnetic moments of *μ*_eff_=3.16 and *μ*_eff_=1.91 μ_B_ for [Ni(bpzCal)] (two unpaired electrons) and [Cu(bpzCal)] (one unpaired electron) respectively, as determined by the Evans method on CD_2_Cl_2_ solutions of the complexes. Therefore only [Cu(bpzCal)] is X‐band EPR active (Figure S10 in the Supporting Information) in contrast to [Ni(bpzCal)] for which the large zero‐field splitting (ZFS) of the *S*=1 spin state results in the absence of any observable signal.

Cyclovoltammetric (CV) measurements on both complexes surprisingly revealed variations in the electrochemical behavior: in contrast to [Ni(bpzCal)], which exhibits only one quasi‐reversible oxidation wave at *E*_1/2_=130 mV, [Cu(bpzCal)] can be oxidized a second time at a potential of ^2^ *E*=670 mV (^1^ *E*_1/2_=110 mV) although this event is highly irreversible (Figure 4). To understand the origin of the oxidation (metal‐ or ligand‐based) and the differing behavior, the corresponding complex of the redox‐inert metal zinc [Zn(bpzCal)] was analyzed. This compound also exhibits oxidation events in the CV in a similar region. However, in contrast to the cyclic voltammograms of the lighter analogues, the zinc complex exhibits two quasi‐reversible oxidation waves (^1^ *E*_1/2_=135, ^2^ *E*_1/2_=340 mV) separated by only 205 mV (Figure 4). This indicates that the redox events found for the previously mentioned complexes likely are not metal‐based but involve the phenolate functions.

**Figure 4:**
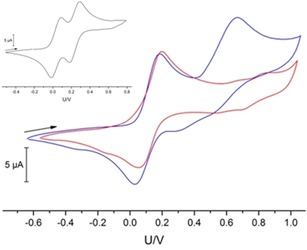
Cyclic voltammograms recorded vs. Fc/Fc^+^ (100 mm [*n*Bu_4_N]PF_6_, Au/Pt/Pt electrodes, 100 mVs^−1^) of [Ni(bpzCal)] (red) and [Cu(bpzCal)] (blue) in CH_2_Cl_2_ (1 mm) at 293 K. Inset: cyclic voltammogram of [Zn(bpzCal)] (same conditions).

The different appearances of the three CVs can then be explained by the different degrees of electronic delocalization and communication after the first oxidation event: the electronic communication between the oxidized and the remaining second phenolate cannot be mediated effectively by the central ion in the case of zinc because of its completely filled d^10^ configuration, leading to two oxidations at potentials that do not differ significantly;^12^ indeed there are also examples reported in which both electrons are removed simultaneously.^13^ The communication can be facilitated by the open‐shell 3d metal ions nickel(II) and copper(II), so that the removal of a second electron is affected more significantly.^14^ Hence, in the case of [Cu(bpzCal)] this second oxidation is shifted to much higher potentials and is irreversible in nature. This shift will be even more pronounced in the case of [Ni(bpzCal)] so that it is not observable anymore in the potential window of the used solvent.

To confirm the inferences made above spectroscopically, we performed spectroelectrochemical measurements. When collecting UV/Vis spectra while going through the first oxidation waves of the neutral complexes with a low scan rate, the formation of new bands was observed (Figure 5).

**Figure 5:**
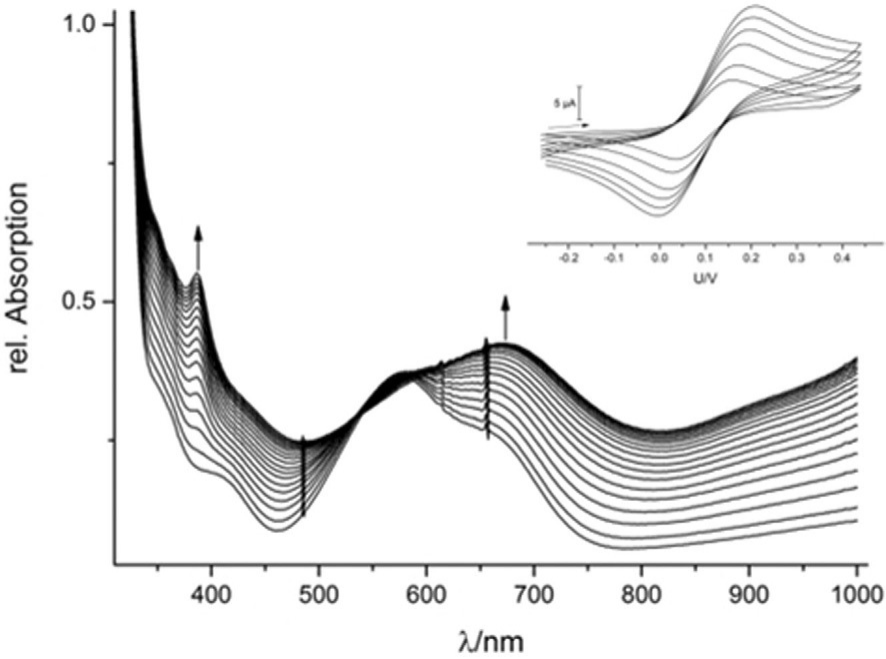
UV/Vis spectral changes observed for 1 mm CH_2_Cl_2_ solutions of [Cu(bpzCal)] during cyclic voltammetry, while moving through the first oxidation wave with 5 mVs^−1^ at 293 K. Inset: cyclic voltammograms of [Cu(bpzCal)] at different scan rates (conditions see Figure 4).

The electronic absorption spectra of the oxidized species have a new strong absorption band around 400 nm in common (402 for [Ni(bpzCal)]^+^ and 392 nm for [Cu(bpzCal)]^+^). Given that [Zn(bpzCal)]^+^ also shows such a band at 401 nm, these absorptions (Figures S8 and S9 in the Supporting Information) can be assigned to the π–π* transition of a phenoxyl radical.^15^

For further investigations, we headed towards chemical oxidation of the complexes. AgSbF_6_ (*E*_1/2_=650 mV vs. Fc/Fc^+^)^16^ proved to be a suitable one‐electron oxidant to realize the first oxidation. Addition of one equivalent of the silver salt to solutions of dark‐blue [Cu(bpzCal)] or deep‐red [Ni(bpzCal)] in CH_2_Cl_2_ caused an immediate color change to dark‐green or dark‐orange, respectively, accompanied by precipitation of elemental silver. These oxidation products gave UV/Vis spectra identical to those of the electrochemically generated species.

In previous extensive studies resonance Raman (rR) spectroscopy has proven a powerful tool for the detection of phenoxyl radicals. When CH_2_Cl_2_ solutions of the oxidized compounds are excited with a 413 nm laser, which matches the electronic transitions of the radical at around 400 nm well, especially the modes ν_7a_ and ν_8a_ of the phenoxyl radicals are enhanced (Figure 6).

**Figure 6:**
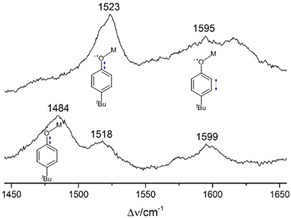
Resonance Raman spectra (Kr^+^ laser, *λ*_exc_=413 nm, 5 mW) of [Ni(bpzCal)]^+^ (top) and [Cu(bpzCal)]^+^ (bottom) in CH_2_Cl_2_ (4 mm) at 203 K.

In contrast to the nickel derivative, [Cu(bpzCal)]^+^ shows a strong band at 1484 cm^−1^, which can be assigned to a phenolate vibration by comparison with the reduced complex (Figure S6 in the Supporting Information). This points towards a localization of the radical in this case. Clearly, the nickel compound features a more delocalized electronic structure and therefore lacks a phenolate band in its rR spectrum. Both complexes exhibit a band at around 1520 cm^−1^ assignable to the ν_7a_ mode of the C−O vibration. The frequencies of these bands are somewhat higher than those found for other known metal‐coordinated phenoxyl radical species, suggesting a larger contribution of the quinonoid canonical form in our case. The ν_8a_ mode of the C_ortho_−C_meta_ vibration appears at around 1600 cm^−1^ in both cases fitting well with reported values.^17^

Galactose oxidase in its active form has a diamagnetic ground state through antiferromagnetic coupling of the ligand‐radical spin with the unpaired d‐electron of the copper(II) metal ion.^18^ Therefore, it was of interest to determine which spin state the phenoxyl radical complexes described above adopt. Given the limited stability of the oxidized species in the solid state, no reliable SQUID data could be obtained. However, magnetic susceptibilities in solution could be determined by using Evans method: [Ni(bpzCal)]^+^ has a magnetic moment of *μ*_eff_=3.67 μ_B_ fitting well with the expected value for three unpaired electrons, thereby suggesting a *S*=3/2 spin state. In contrast [Cu(bpzCal)]^+^ has a moment of *μ*_eff_=2.66 μ_B_ which is close to two unpaired electrons of a *S*=1 spin state, definitely excluding a diamagnetic ground state in our case.

To further corroborate the actual spin states, X‐band EPR measurements of frozen solutions were performed: in contrast to its neutral precursor, [Ni(bpzCal)]^+^ is EPR active and exhibits a rhombic signal that was simulated best with *g_x_*=2.36, *g*_y_=2.34, and *g*_z_=2.24 (Figure S12 in the Supporting Information). The *g*‐factors of typical Ni^III^ species are usually somewhat lower at around 2.1, thus a metal centered oxidation in our case can again be excluded. The signals are not well resolved, which may be attributed to the general difficulty of collecting EPR spectra of *S*=3/2 systems at liquid‐nitrogen temperatures. The original EPR signal of [Cu(bpzCal)] is attenuated in the course of the oxidation leaving only a residual signal for an organic radical (*g*≈2) together with one for copper(II) (Figure S11). They account for less than 10 % of the total spin concentration, likely originating from a disproportionation reaction yielding Cu^II^ and a twofold oxidized species. [Zn(bpzCal)]^+^ is the most unstable complex of the series, thus only traces (≈10 %) of an organic radical with *g*_iso_=2.00 were detected by EPR (Figure S13).

Given that no crystals could be grown to investigate the molecular structures of these compounds due to the high instability of the oxidized complexes, DFT calculations were performed to gain insights into possible structures and spin states: for [Cu(bpzCal)]^+^ two isomers were found, one with the same symmetry as its neutral precursor and one asymmetric version, both in a singlet and a triplet state (Figure 7). With exception of the symmetric closed‐shell singlet state, all structures and states are very close in energy (Table S1 in the Supporting Information), with the asymmetric triplet as ground state, matching the results described above.

**Figure 7:**
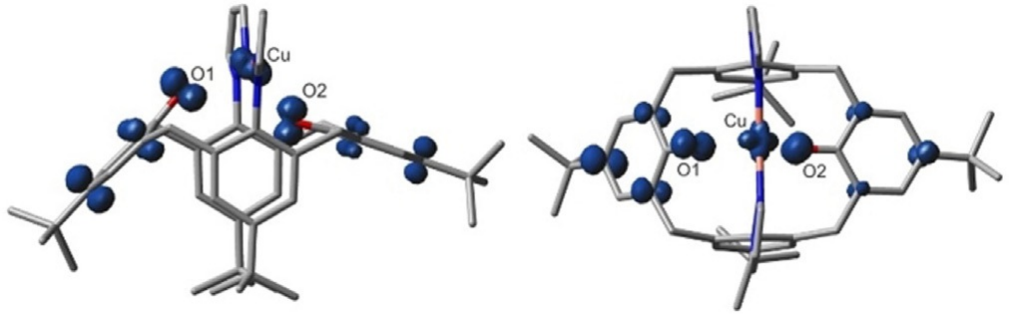
Optimized molecular structures and Mulliken spin‐density distributions of the asymmetric triplet state of [Cu(bpzCal)]^+^. Hydrogen atoms are omitted for clarity.

Both in the symmetric and the asymmetric isomer of [Cu(bpzCal)]^+^ the Cu atom was then replaced by Ni followed by re‐optimization. Given that Ni has one electron less than Cu, doublet and quartet states result. For the doublet state, a symmetric and an asymmetric isomer were found, however, for the quartet state all optimizations resulted in a symmetric structure, which is the ground state of the molecule (Table S2 and Figures S19–S21 in the Supporting Information), which is in agreement with the experimental findings.

Hence, from the above data it becomes very clear that a complex with copper(II) ion in a coordination sphere resembling the one in the active site of the GO can be generated and that it can be oxidized at the phenolate donor to yield a radical complex with a triplet ground state. This complex compares well with the active state of the GO, with the difference that the latter has a singlet ground state. To that extent it was now of much interest to test the reactivity of our complexes towards a primary alcohol.

Accordingly, chemically generated solutions of [Cu(bpzCal)]^+^ and, for comparison, also [Ni(bpzCal)]^+^ in CH_2_Cl_2_ were treated with benzyl alcohol as the model substrate. The formation of the two‐electron oxidation product benzaldehyde was indeed detected through ^1^H NMR spectroscopy; however, unlike in case of the enzymatic paragon, formation of only half an equivalent of the aldehyde per equivalent complex was observed (Scheme 2).

**Scheme 2:**
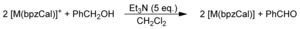
Benzyl alcohol oxidation with [M(bpzCal)]^+^ (M=Ni, Cu).

Although in the enzymatic catalytic cycle the Cu^II^ center performs the second oxidation step (→Cu^I^) its oxidation potential in the complex is obviously not sufficiently positive, so that a second equivalent of the complex is needed.^19^ Indeed, the CV of [Cu(bpzCal)] exhibits a redox event assigned to the Cu^2+^/Cu^+^ couple (Figure S7 in the Supporting Information) at a potential (^red^ *E*=−1.53 V) that is not suitable to perform the alcohol oxidation.

In conclusion, we reported here a model of the GO with a unique biomimetic donor sphere around the copper center that is provided by a calixarene framework. In contrast to most model complexes known so far [Cu(bpzCal)] features a nonplanar structure that resembles the one of the His_2_–Tyr_2_ core found for the enzyme, which, however, coordinates in the active state an additional water molecule and is able to release one of the Tyr donors upon protonation. Like the enzyme, the model can be singly oxidized to yield a phenoxyl radical coordinating the copper center, mimicking the active state of the GO albeit with a different spin state. The latter, however, is not decisive for the reactivity: the oxidized complex was capable of converting benzyl alcohol to the corresponding aldehyde, like the GO does, but through a different mechanism involving two equivalents of the complex. To mimic the two‐electron oxidation more faithfully, future attempts will focus on the stabilization of the copper(I) state through the ligand system so that the copper(II) state becomes more oxidizing.

## Conflict of interest

The authors declare no conflict of interest.

## Supporting information

As a service to our authors and readers, this journal provides supporting information supplied by the authors. Such materials are peer reviewed and may be re‐organized for online delivery, but are not copy‐edited or typeset. Technical support issues arising from supporting information (other than missing files) should be addressed to the authors.

Supplementary
