# Supplementary material for: Oxygen‐Depleted Calixarenes as Ligands for Molecular Models of Galactose Oxidase
Source: Chemistry. 2019 Sep 19;25(58):13285–9. doi: 10.1002/chem.201903820 (PMC6857004; doi:10.1002/chem.201903820)
Supplement: Supplementary file 1 — Supplementary [file CHEM-25-13285-s001.pdf]

CHEMISTRY
A European Journal

Supporting Information

Oxygen-Depleted Calixarenes as Ligands for Molecular Models of Galactose Oxidase

Matthias Keck,[a] Santina Hoof,[a] Christian Herwig,[a] Arkadi Vigalok,[b] and Christian Limberg*[a]

chem_201903820_sm_miscellaneous_information.pdf

Table of Contents

Experimental Section	S3
^{1}H NMR Spectra	S7
IR/rR Spectra	S8
(Spectro-)Electrochemistry	S10
EPR Spectra	S11
X-Ray Crystallographic Information	S13
Density Functional Calculations	S16
References	S26

Experimental Section

General Considerations. All manipulations were carried out in an argon atmosphere using conventional Schlenk techniques or in gloveboxes with either dinitrogen or argon atmospheres maintained below 1 ppm of O_2 and H_2O. Glassware was heated under vacuum using a heat gun at 650 °C prior to use. All solvents used for reactions were either dried with a MBraun solvent purification system (SPS) (THF was additionally distilled from Solvona®) or by using standard literature methods. Degassing was performed by bubbling argon through the solvent for 20 minutes. Deuterated solvents for NMR were dried by storing over 3 Å molecular sieves, degassed by freeze-pump-thaw technique (3 cycles) and stored in a glove box. NMR spectra were recorded on a Bruker AVANCE II 300 spectrometer. Chemical shifts are referenced to the residual proton signal of the solvent. Magnetic susceptibility measurements were carried out using the Evan's NMR method.[1] IR spectra were recorded in an argon filled glovebox on a Bruker ALPHA spectrometer with an ATR sampling unit. EPR samples were recorded on a Benchtop Magnettech MiniScope MS 5000 spectrometer. Resonance Raman spectra were recorded on a Horiba Jobin-Yvon LabRAM HR800 confocal Raman spectrometer equipped with a Horoiba Symphony II CCD detector and for excitation a Coherent Innova 400 Kr^+-laser was used. The samples in standard 5 mm NMR tubes were cooled with a Bruker R 495 cryostat. Cyclic voltammograms (CVs) were recorded in an argon filled glovebox using a PalmSens EmStat Blue potentiostat. A conventional one-compartment three-electrode cell was equipped with a Au-disk working electrode, a Pt-wire as the auxiliary electrode and a Pt-wire as pseudo-reference. All data were referenced to the Fc/Fc^+ couple at the end of each measurement. For spectroelectrochemical measurements a Gamry Instruments Reference 600 potentiostat/galvanostat was used. A SUPRASIL Quartz cell from Hellma Analytics with a 1 mm path length was used and equipped with a Pt-gauze working electrode (1 cm^2), a Pt-wire as the auxiliary electrode and a Pt-wire as pseudo-reference. UV-vis spectra were collected under an argon atmosphere with an Agilent 8453 UV-vis spectrophotometer. Electrospray ionization (ESI) mass spectra were obtained on an Agilent Technologies 6210 Time-of-Flight LC-MS instrument. Elemental analyses were performed with a HEKA Euro 3000 elemental analyser.

$ZnEt_2$ (1 M in *n*-hexane), $AgSbF_6$ (anhydrous), benzyl alcohol (anhydrous) and triethylamine (anhydrous) were purchased from Sigma-Aldrich and used as received. H_2(bpz-Cal[4]) [2], $[Ni(NPh_2)_2]_2$ [3] and $Cu(dmap)_2$ [4] were prepared according to literature procedures.

Synthesis of [Ni(bpzCal)]. [H_2(bpzCal)] (50.0 mg, 66.8 μmol) was dissolved in THF (10 mL), a solution of [Ni(NPh_2)$_2$]$_2$ (26.4 mg, 33.4 μmol, 0.5 eq.) in THF (10 mL) was added and the mixture heated for 16 h under reflux. After cooling to room temperature, all volatiles were removed from the deep red solution were removed in vacuo and the obtained solid was washed with *n*-hexane (3×5 mL). After drying in high vacuum, [Ni(bpzCal)] was isolated as brown solid in 62% yield (33.4 mg, 41.5 μmol).

^{1}H NMR (THF-d_8, 300 MHz): δ [ppm] = 100.51 (s, 2H), 55.95 (s, 2H), 26.82 (s, 4H), 21.33 (s, 2H), 15.15 (s, 4H), 9.33 (s, 4H), 6.39 (s, 18H), 4.00 (s, 4H), 2.91 (s, 18H).

ATR-IR (solid): ν [cm^{-1}] = 2953 (m), 2906 (w), 2864 (w), 1598 (w), 1514 (w), 1475 (s), 1427 (m), 1405 (m), 1360 (w), 1323 (s), 1308 (m), 1292 (w), 1213 (m), 1168 (w), 1114 (w), 1064 (m), 1030 (w), 965 (w), 921 (w), 903 (w), 870 (m), 834 (w), 814 (w), 800 (w), 755 (s), 633 (w), 620 (w), 561 (w), 547 (w), 530 (w), 511 (w), 429 (w).

ESI-MS (CH_2Cl_2, +MS): *m/z* = 805.3974 $[M+H]^+$ (calc.: 805.3991), 827.3789 $[M+Na]^+$ (calc.: 827.3811).

EA for $C_{50}H_{58}N_4O_2Ni$ (805.71 g mol^{-1}) [%]: calc.: C 74.53, H 7.26, N 6.95; found: C 74.36, H 7.61, N 6.71.

Magnetic moment (Evans, CD_2Cl_2, 300 MHz, RT): μ_{eff} = 3.16 μ_B.

Crystals suitable for X-ray diffraction analysis were grown by slow evaporation of the volatiles from a solution of [Ni(bpzCal)] in THF at room temperature.

Synthesis of [Cu(bpzCal)]. [H_2(bpzCal)] (50.0 mg, 66.8 μmol) was dissolved in THF (10 mL), a solution of Cu(dmap)$_2$ (17.9 mg, 66.8 μmol, 1 eq.) in THF (10 mL) was added and the mixture heated for 16 h under reflux. After cooling to room temperature, all volatiles were removed from the deep blue solution were removed in vacuo and the obtained solid was washed with *n*-hexane (3×5 mL). After drying in high vacuum, [Cu(bpzCal)] was isolated as purple solid in 70% yield (37.8 mg, 46.6 μmol).

ATR-IR (solid): ν [cm^{-1}] = 2953 (m), 2907 (w), 2864 (w), 1600 (w), 1515 (w), 1456 (s), 1427 (m), 1405 (m), 1359 (w), 1299 (s), 1246 (w), 1206 (m), 1181 (w), 1116 (w), 1089 (w), 1069 (m), 1031 (w), 1012 (w), 966 (w), 944 (w), 918 (w), 903 (w), 883 (w), 868 (m), 827 (w), 815 (w), 800 (w), 755 (s), 670 (w), 633 (w), 620 (w), 555 (w), 543 (w), 527 (w), 505 (w), 429 (w).

ESI-MS (CH_2Cl_2, +MS): *m/z* = 810.3906 $[M+H]^+$(calc.: 810.4034), 832.3731 $[M+Na]^+$(calc.: 832.3853).

EA for $C_{50}H_{58}N_4O_2Cu$ (810.57 g mol^{-1}) [%]: calculated: C 74.09, H 7.21, N 6.91; found: C 74.10, H 7.58, N 6.76.

Magnetic moment (Evans, CD_2Cl_2, 300 MHz, RT): μ_{eff} = 1.91 μ_B.

Crystals suitable for X-ray diffraction analysis were grown by slow evaporation of the volatiles from a solution of [Cu(bpzCal)] in THF at room temperature.

Synthesis of [Zn(bpzCal)]. *This modified synthesis is based on a known literature procedure*[2] [H_2(bpz-Cal)] (50.0 mg, 66.8 μmol) was dissolved in THF (10 mL), a solution of $ZnEt_2$ (1 M in *n*-hexane, 66.8 μL, 66.8 μmol, 1 eq.) was added and the mixture heated for 16 h under reflux. After cooling to room temperature, all volatiles were removed from the slightly yellow solution were removed in vacuo and the obtained solid was washed with *n*-hexane (3×5 mL). After drying in high vacuum, [Zn(bpzCal)] was isolated as colourless solid in 51% yield (27.5 mg, 33.9 μmol).

^{1}H NMR (THF-d_8, 300 MHz): δ [ppm] = 8.16 (d, $^2J_{HH}$ = 1.7 Hz, 2H, $^5\mathbf{H}_{pz}$), 7.88 (d, $^2J_{HH}$ = 1.9 Hz, 2H, $^3\mathbf{H}_{pz}$), 6.96 (s, 4H, C$\mathbf{H}_{Ar}$), 6.87 (s, 4H, C$\mathbf{H}_{Ar}$), 6.66 (t, $^2J_{HH}$ = 2.3 Hz, 2H, $^4\mathbf{H}_{pz}$), 3.24 (d, $^1J_{HH}$ = 13.1 Hz, 4H, ^{exo}C$\mathbf{H}_2$), 3.11 (d, $^1J_{HH}$ = 13.3 Hz, 4H, ^{endo}C$\mathbf{H}_2$), 1.28 (s, 18H, C(C$\mathbf{H}_3$)$_3$), 0.98 (s, 18H, C(C$\mathbf{H}_3$)$_3$).

ATR-IR (solid): ν [cm^{-1}] = 2953 (m), 2906 (w), 2865 (w), 1598 (w), 1515 (w), 1476 (s), 1428 (w), 1405 (m), 1392 (w), 1361 (w), 1319 (s), 1206 (m), 1181 (w), 1116 (w), 1066 (m), 1055 (w), 1028 (w), 964 (w), 922 (w), 904 (w), 868 (m), 838 (w), 812 (w), 799 (w), 762 (s), 753 (s), 633 (w), 621 (w), 599 (w), 562 (w), 549 (w), 529 (w), 510 (w), 429 (w).

ESI-MS (CH_2Cl_2, +MS): *m/z* = 811.3894 $[M+H]^+$ (calc.: 811.3929), 833.3706 $[M+Na]^+$ (calc.: 833.3748), 849.3446 $[M+K]^+$ (calc.: 849.3488).

EA for $C_{50}H_{58}N_4O_2Zn$ (812.40 g mol^{-1}) [%]: calculated: C 73.92, H 7.20, N 6.90; found: C 73.80, H 7.26, N 6.81.

Crystals suitable for X-ray diffraction analysis were grown by slow evaporation of the volatiles from a solution of [Zn(bpzCal)] in THF at room temperature.

Chemical Oxidation of [Ni(bpzCal)], [Cu(bpzCal)] and [Zn(bpzCal)]

[Ni(bpzCal)], [Cu(bpzCal)] or [Zn(bpzCal)] (1 eq.) was dissolved in CH_2Cl_2, a solution of $AgSbF_6$ (1 eq.) in CH_2Cl_2 was added and the mixture stirred for 5 min followed by filtration.
These solutions have always been freshly prepared and used immediately for the different analytical techniques (in the required concentrations) or for reactivity tests.

Reactivity of [Ni(bpzCal)] and [Cu(bpzCal)] towards benzyl alcohol

To solutions of $[Ni(bpzCal)]^+$ or $[Cu(bpzCal)]^+$ (1 eq.) in CH_2Cl_2 prepared as stated above was added a solution of benzyl alcohol dissolved in CH_2Cl_2 (x eq.) pre-treated with triethylamine (5 eq.) at room temperature and the mixture stirred for 5 min.
Product analyses and quantifications (1,3,5-Tribromobenzene as standard) have been performed by ^1H NMR spectroscopy.

Equivalents of substrate	0.5	1.0	2.0
Determined yield [%]	94	45	23

^{1}H NMR Spectra

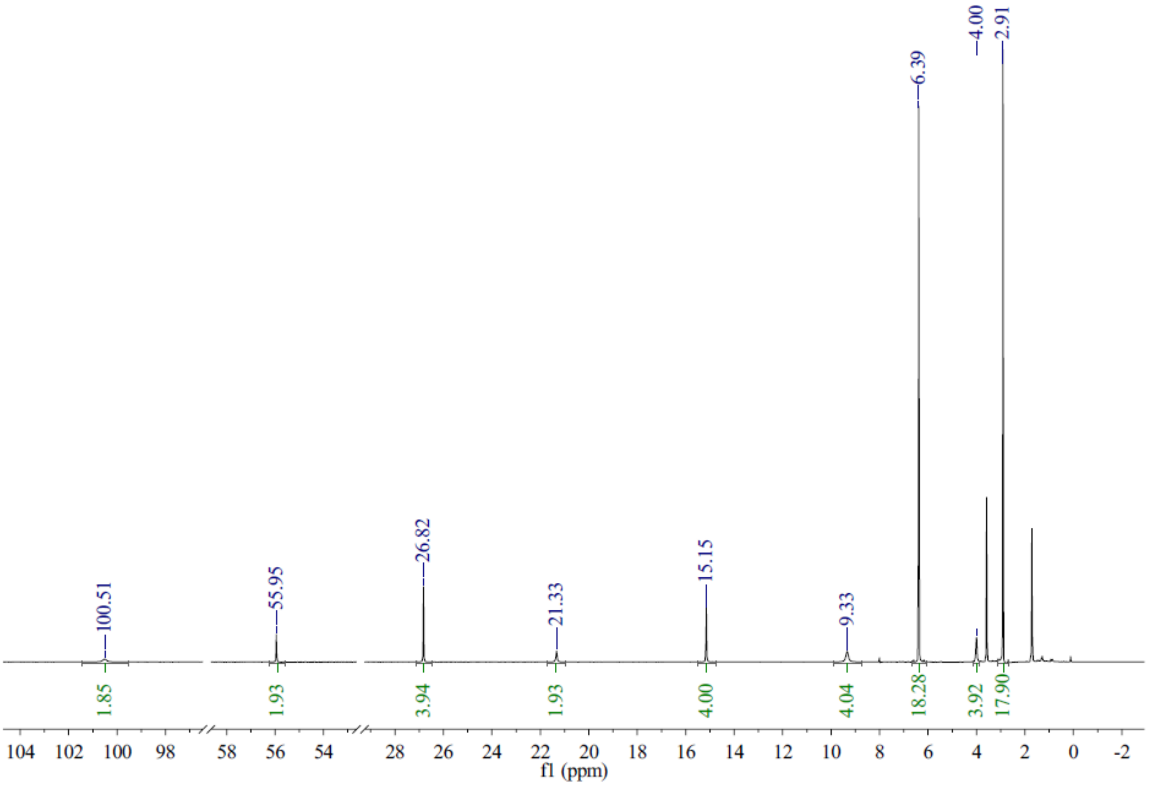

Figure S1 – ^{1}H NMR spectrum (300 MHz, THF-d_8, 293 K) of [Ni(bpzCal)].

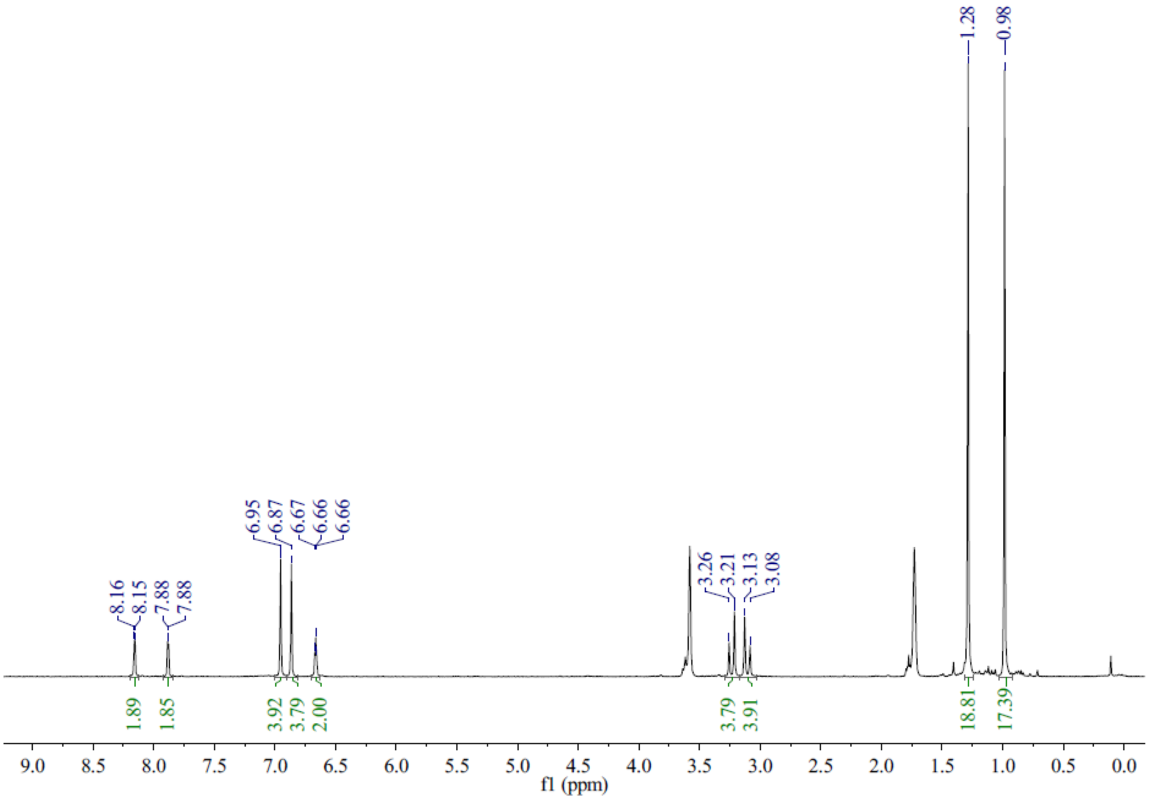

Figure S2 – ^{1}H NMR spectrum (300 MHz, THF-d_8, 293 K) of [Zn(bpzCal)].

IR/rR Spectra

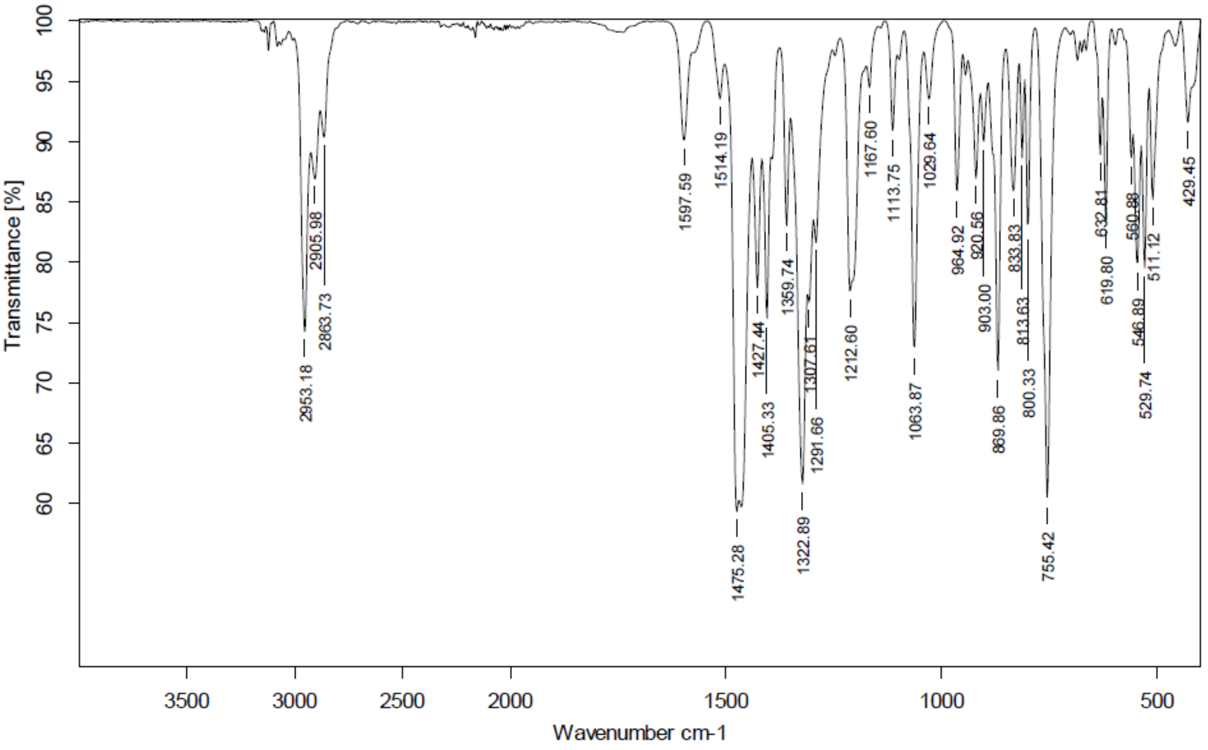

Figure S3 – ATR-IR spectrum of [Ni(bpzCal)] (solid).

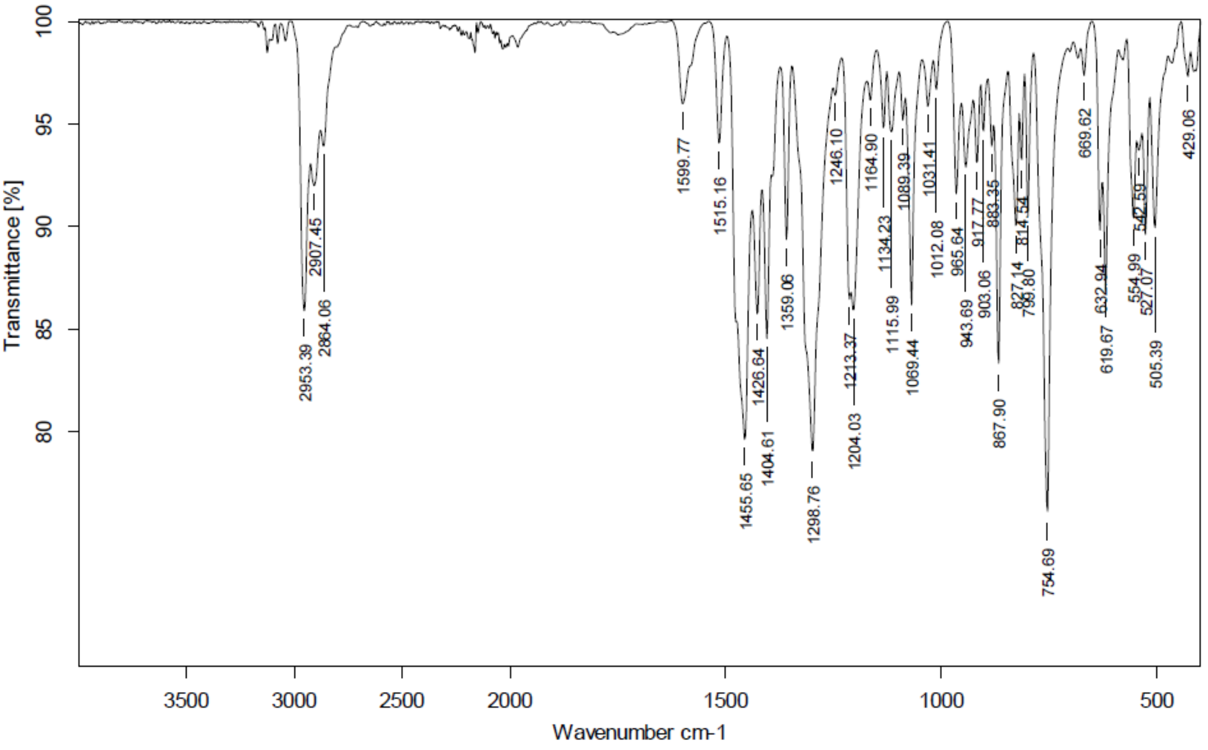

Figure S4 – ATR-IR spectrum of [Cu(bpzCal)] (solid).

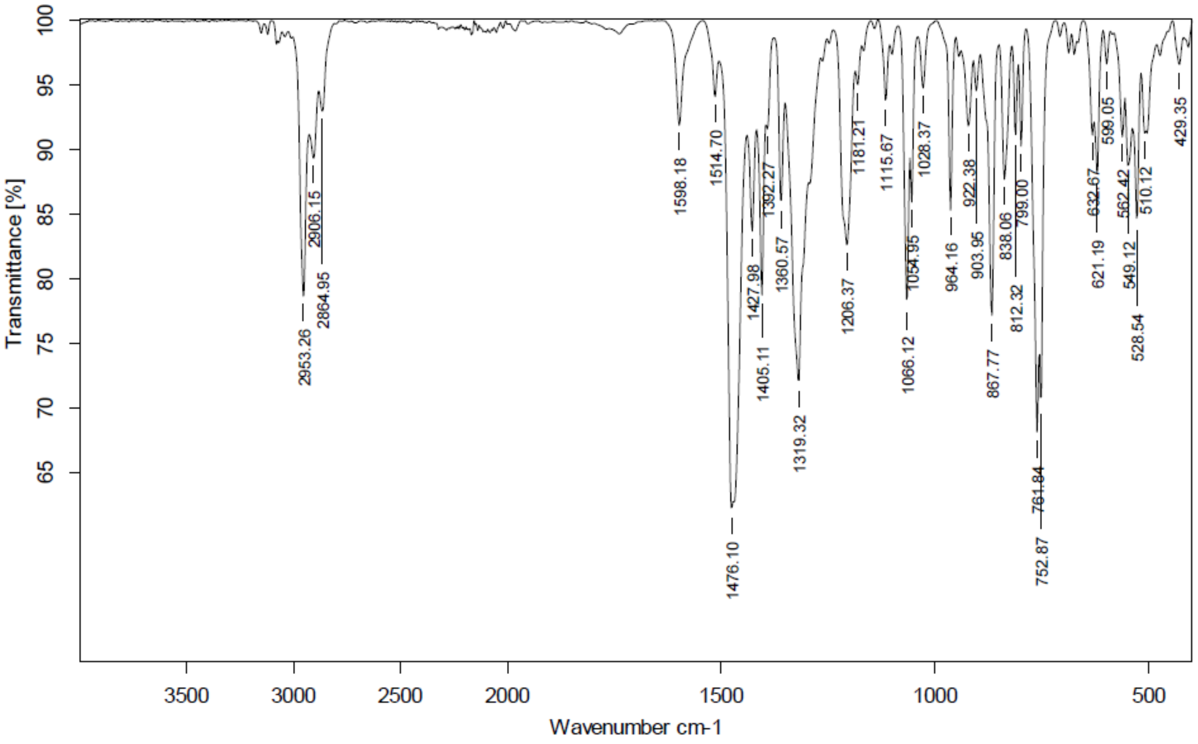

Figure S5 – ATR-IR spectrum of [Zn(bpzCal)] (solid).

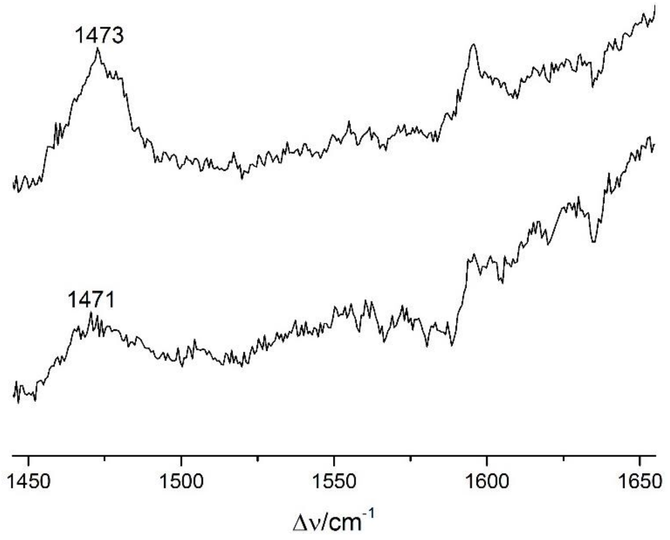

Figure S6 – rR spectra (Kr^+-laser, λ_{exc} = 413 nm, 5 mW) of [Ni(bpzCal)] (top) and [Cu(bpzCal)] (bottom) in CH_2Cl_2 (4 mM) at 203 K.

(Spectro-)Electrochemistry

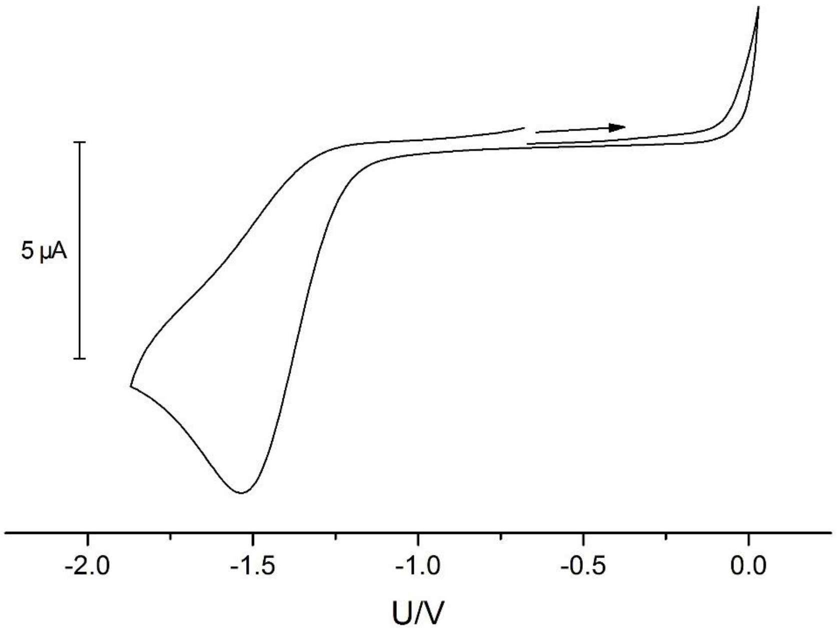

Figure S7 – Voltammogram (Au/Pt/Pt, 100 mM $TBAPF_6$ in CH_2Cl_2, vs. Fc/Fc^+, 100 mVs^{-1}) of [Cu(bpzCal)] (1 mM) at 293 K.

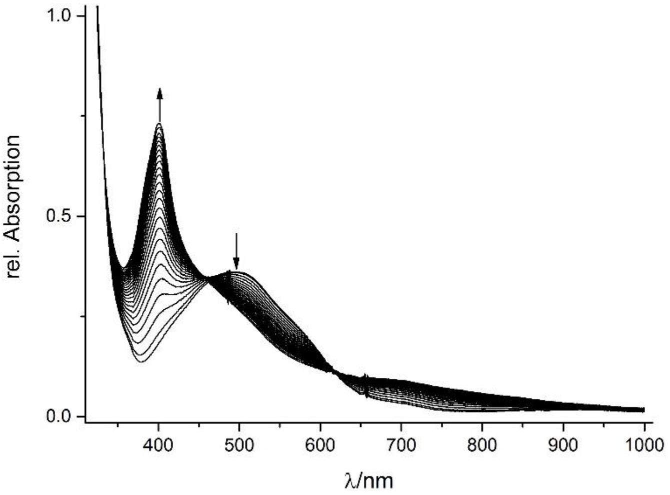

Figure S8 – UV-vis spectral changes of [Ni(bpzCal)] during cyclic voltammetry, while moving through the first oxidation wave with 5 mVs^{-1} in CH_2Cl_2 (1 mM) at 293 K.

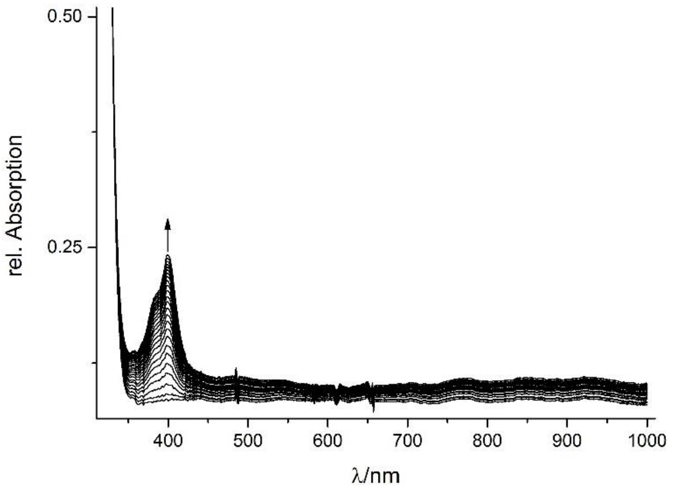

Figure S9 – UV-vis spectral changes of [Zn(bpzCal)] during cyclic voltammetry, while moving through the first oxidation wave with 5 mVs^{-1} in CH_2Cl_2 (1 mM) at 293 K.

EPR Spectra

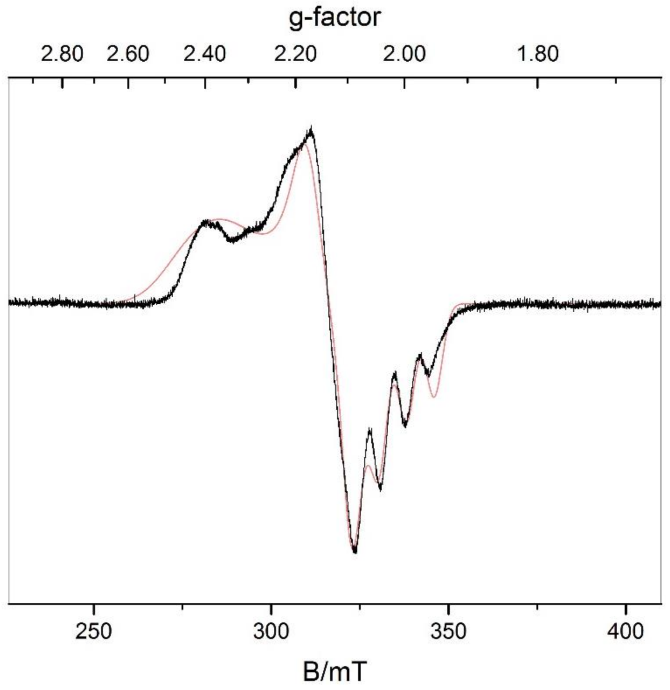

Figure S10 – Experimental X-band EPR spectrum of [Cu(bpzCal)] in CH_2Cl_2 (1 mM) at 77 K (black) & simulation thereof (red).

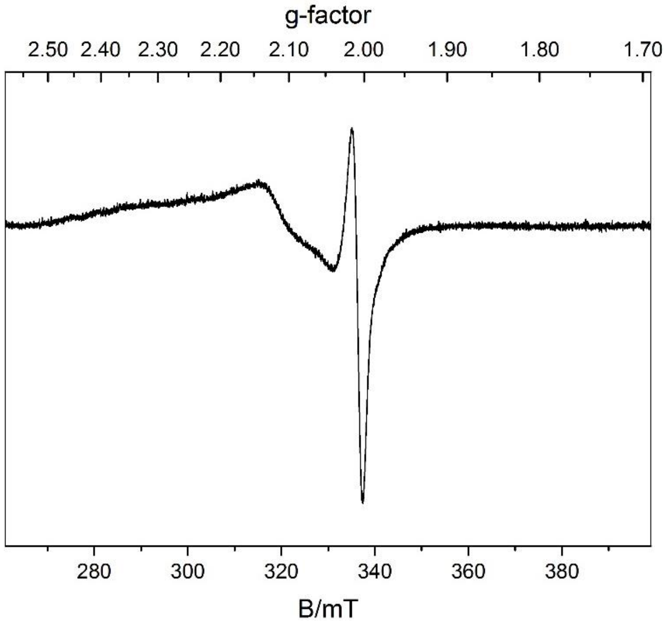

Figure S11 – Experimental X-band EPR spectrum of $[Cu(bpzCal)]^+$ in CH_2Cl_2 (1 mM) at 77 K.

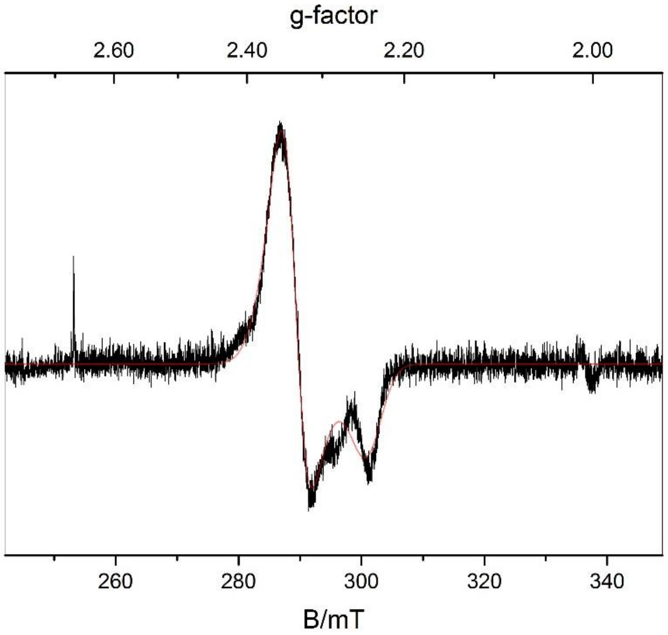

Figure S12 – Experimental X-band EPR spectrum of $[Ni(bpzCal)]^+$ in CH_2Cl_2 (1 mM) at 77 K (black) & simulation thereof (red).

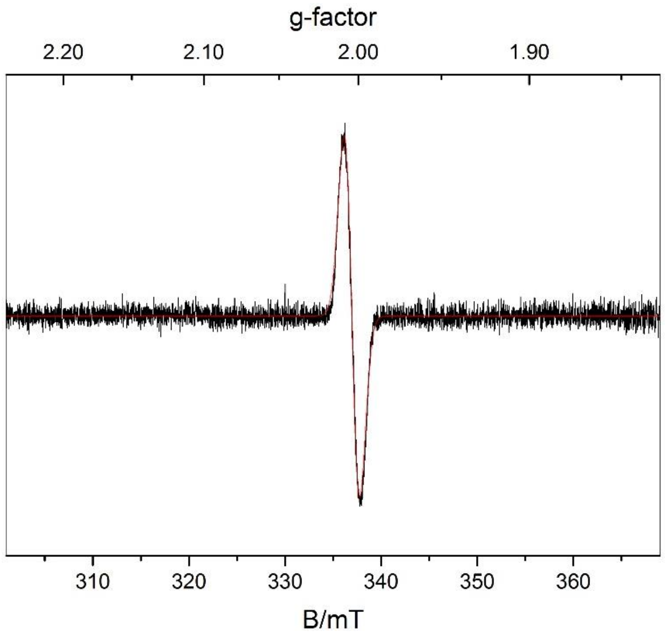

Figure S13 – Experimental X-band EPR spectrum of $[Zn(bpzCal)]^{+}$ in CH_2Cl_2 (1 mM) at 77 K (black) & simulation thereof (red).

X-Ray crystallographic information

The data collections were performed with a BRUKER D8 VENTURE area detector with Mo-Kα radiation (λ = 0.71073 Å). Multi-scan absorption corrections implemented in SADABS[5] were applied to the data. The structures were solved by intrinsic phasing method (SHELXT-2013)[6] and refined by full matrix least square procedures based on F2 with all measured reflections (SHELXL-2014 in the graphical user interface SHELXle)[7] with anisotropic temperature factors for all non-hydrogen atoms, except the solvent of crystallisation in complex ([Cu(bpzCal)]). All hydrogen atoms were added geometrically and refined by using a riding model. The SQUEEZE function implemented in PLATON was used for complex [Zn(bpzCal)].[8] CCDC 1939607 ([Ni(bpzCal)]), 1938437 ([Cu(bpzCal)]), 1938438 ([Zn(bpzCal)]) contain the supplementary crystallographic data for this paper. These data can be obtained free of charge from The Cambridge Crystallographic Data Centre via www.ccdc.cam.ac.uk/data_request/cif.

Crystallographic Data for [Ni(bpzCal)]:

It has to be mentioned that only low quality crystals could be measured.

$C_{50}H_{58}N_4O_2Ni{\cdot}0.5\ C_4H_8O$, M_r = 841.76, triclinic, *P*-1, red needle, 0.250 x 0.060 x 0.020 mm, *a* = 11.867(2) Å, b = 20.241(4) Å, c = 20.516(4) Å, α = 100.339(9) °, β = 96.211(8)°, γ = 104.682(9) °, *V* = 4628.2(15) Å^3, *Z* = 4, ρ = 1.208 g·cm^{-3}, μ = 0.464 mm^{-1}, Mo-K_α radiation (λ = 0.71073 Å), *T* = 100(2) K, Θ = 2.207 to 25.262°, F_{000} = 1800, reflections collected 42859, reflections unique 16228 [R_{int} = 0.1902], GooF = 1.038, *R* = 0.1413, wR_2 = 0.3277, largest diff. peak and hole 1.340 and −1.135 eÅ^{-3}..

Crystallographic Data for [Cu(bpzCal)]:

$C_{50}H_{58}N_4O_2Cu{\cdot}C_4H_8O$, M_r = 882.64, triclinic, *P*-1, blue plate, 0.260 x 0.070 x 0.030 mm, *a* = 12.0000(7) Å, b = 20.2829(12) Å, c = 22.2227(13) Å, α = 107.066(3) °, β = 90.050(3)°, γ = 101.539(3) °, *V* = 5056.0(5) Å^3, *Z* = 4, ρ = 1.160 g·cm^{-3}, μ = 0.476 mm^{-1}, Mo-K_α radiation (λ = 0.71073 Å), *T* = 100(2) K, Θ = 2.224 to 25.440°, F_{000} = 1884, reflections collected 122682, reflections unique 18560 [R_{int} = 0.0853], GooF = 1.085, *R* = 0.0734, wR_2 = 0.1997, largest diff. peak and hole 2.310 and −1.060 eÅ^{-3}.

Crystallographic Data for [Zn(bpzCal)]:

$C_{50}H_{58}N_4O_2Zn$, M_r = 812.37, triclinic, *P*-1, colorless block, 0.190 x 0.170 x 0.080 mm, *a* = 10.2022(6) Å, b = 15.4886(9) Å, c = 18.6184(10) Å, α = 73.206(2) °, β = 77.243(3)°, γ = 80.029(3) °, *V* = 2728.1(3) Å^3, *Z* = 2, ρ = 0.989 g·cm^{-3}, μ = 0.485 mm^{-1}, Mo-K_α radiation (λ = 0.71073 Å), *T* = 100(2) K, Θ = 2.175 to 28.878°, F_{000} = 864, reflections collected 46671, reflections unique 13658 [R_{int} = 0.0649], GooF = 1.100, *R* = 0.0703, wR_2 = 0.1650, largest diff. peak and hole 0.612 and −0.810 eÅ^{-3}.

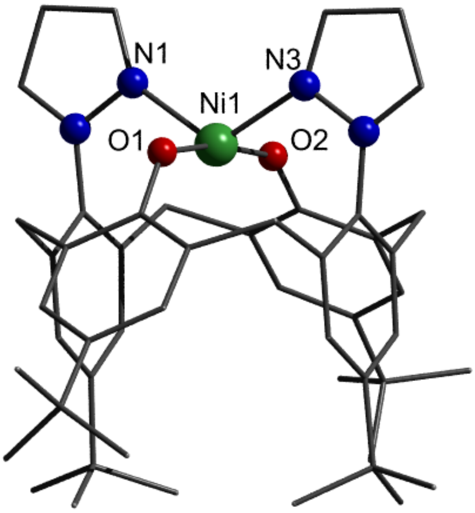

Figure S14 – Molecular structure of [Ni(bpzCal)]. Selected bond lengths [Å] and angles [°]: Ni1-O1 1.876(8), Ni1-O2 1.885(8), Ni1-N1 1.989(10), Ni1-N3 1.994(9), N1-Ni1-N3 116.9(4), O1-Ni1-O2 170.1(3). Hydrogen atoms and co-crystallised solvent molecules are omitted for clarity.

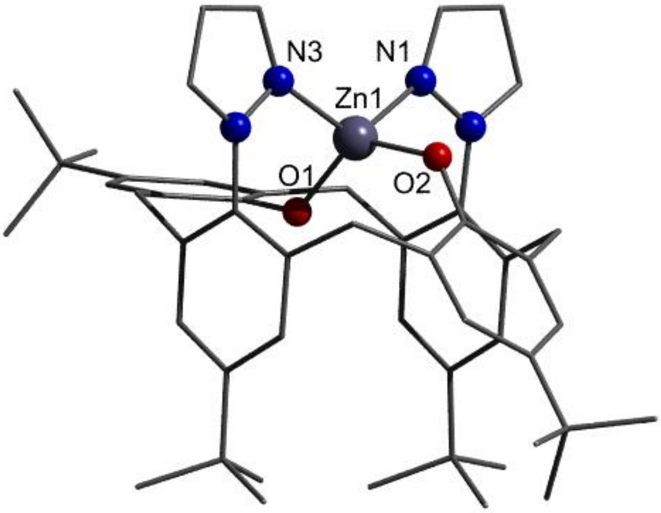

Figure S15– Molecular structure of [Zn(bpzCal)]. Hydrogen atoms and co-crystallised solvent molecules are omitted for clarity. Selected bond lengths [Å] and angles [°]: Zn1-O1 1.885(2), Zn1-O2 1.898(2), Zn1-N1 2.029(3), Zn1-N3 2.024(3), N1-Zn1-N3 120.96(11), O1-Zn1-O2 127.83(9).

Density Functional Calculations

Geometry optimizations were performed in redundant internal coordinates without symmetry restrictions using the Gaussian09 program package.[9] The molecular structure of the neutral complex [Cu(bpzCal)] as determined by X-ray diffraction analysis was used as starting point. The B3LYP functional[10] was employed amended by the D3 version of Grimme's dispersion,[11] together with the Def2-TZVP basis set[12] as implemented in Gaussian. Very tight convergence criteria were chosen for the SCF procedure and a pruned (99,590) "ultrafine" integration grid was used for numerical integrations. Visualisation of molecular structures was accomplished with the program Gauss View (Gaussian, Inc.).

$[Cu(bpzCal)]^+$

**State**	**Relative energy (kJ/mol)**	**Mulliken spin density**
symmetric singlet (closed shell)	58.9	0
symmetric triplet	6.39	Cu: 0.39, O1: 0.26, O2: 0.26, rest of the ligand: 1.09
asymmetric singlet (open shell)	0.62	Cu: 0.21, O1: -0.30, O2: 0.29,<br>rest of the ligand: -0.2 (-1.1 and + 0.9)
asymmetric triplet	0	Cu: 0.26, O1: 0.30, O2: 0.29, rest of the ligand: 1.15

Table S1 – Relative energies and spin density distribution of different isomers and states of $[Cu(bpzCal)]^+$.

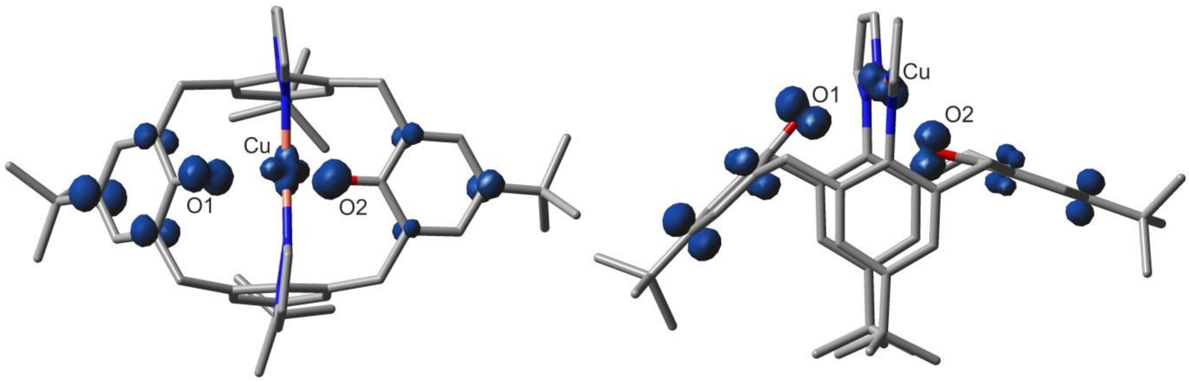

Figure S 16 – Optimised molecular structure and Mulliken spin density distribution of the asymmetric triplet state of [Cu(bpzCal)]$^{+}$. Top and side view. Hydrogen atoms are omitted for clarity.

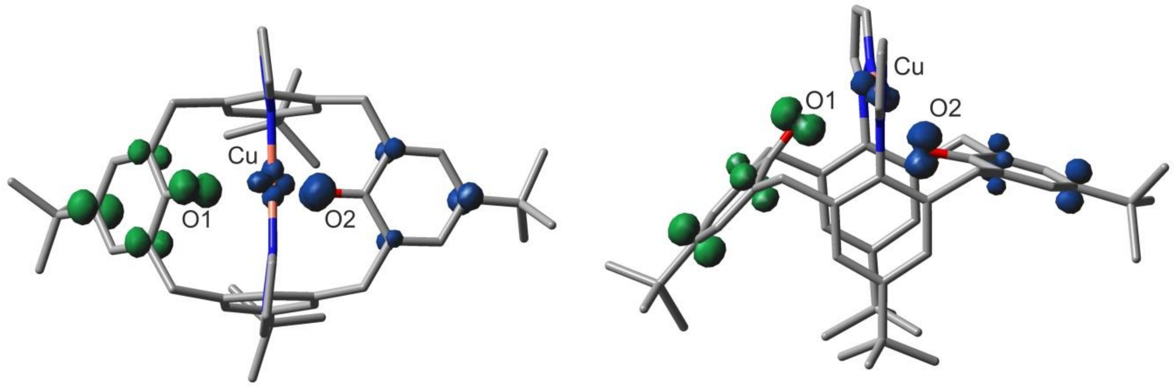

Figure S 17 – Optimised molecular structure and Mulliken spin density distribution of the asymmetric singlet state (open shell) of [Cu(bpzCal)]$^{+}$. Top and side view. Hydrogen atoms are omitted for clarity.

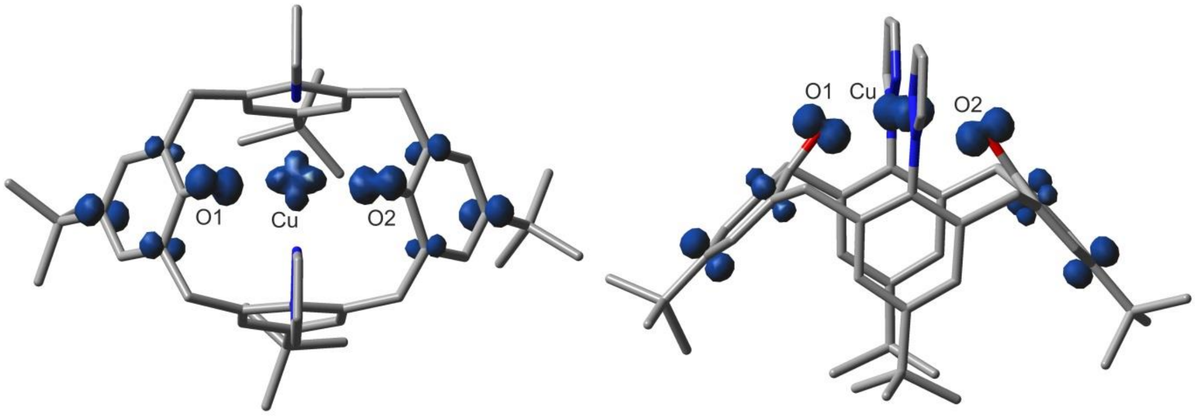

Figure S 18 – Optimised molecular structure and Mulliken spin density distribution of the symmetric triplet state of [Cu(bpzCal)]$^{+}$. Top and side view. Hydrogen atoms are omitted for clarity.

[Ni(bpzCal)]$^+$

State	Relative energy (kJ/mol)	Mulliken spin density
symmetric doublet	38.8	Ni: -0.07, O1: 0.17, O2: 0.17, rest of the ligand: 0.73 (+1.05 and -0.32)
symmetric quartet	0	Ni: 1.74, O1: 0.19, O2: 0.19, rest of the ligand: 0.88
asymmetric doublet	20.1	Ni: 1.60, O1: -0.24, O2: 0.15, rest of the ligand: -0.51 (-0.98 and +0.47)

Table S2 – Relative energies and spin density distribution of different isomers and states of [Ni(bpzCal)]$^+$.

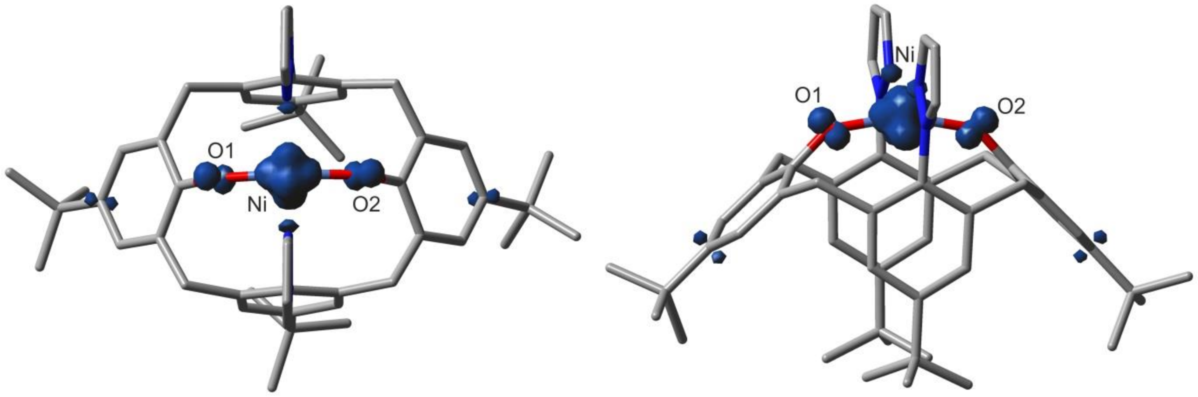

Figure S 19 – Optimised molecular structure and Mulliken spin density distribution of the symmetric quartet state of [Ni(bpzCal)]$^+$. Top and side view. Hydrogen atoms are omitted for clarity.

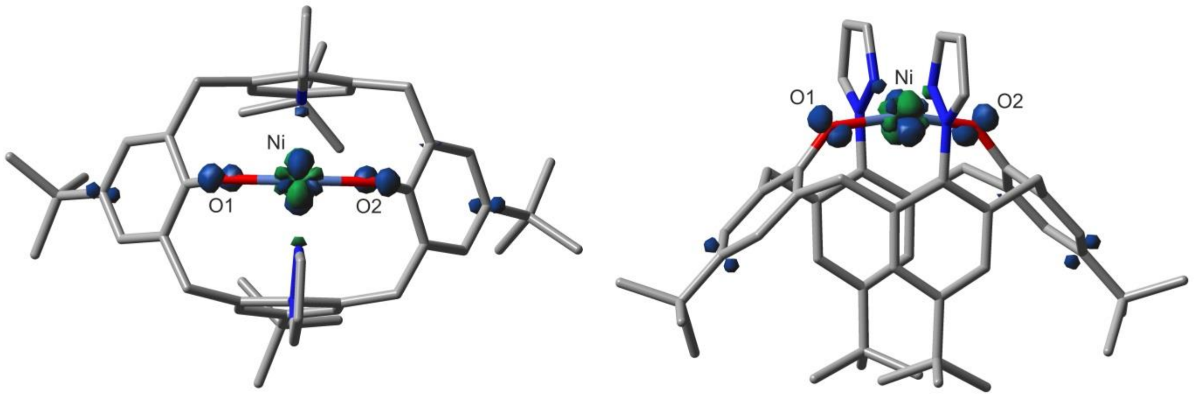

Figure S 20 – Optimised molecular structure and Mulliken spin density distribution of the symmetric doublet state of [Ni(bpzCal)]$^{+}$. Top and side view. Hydrogen atoms are omitted for clarity.

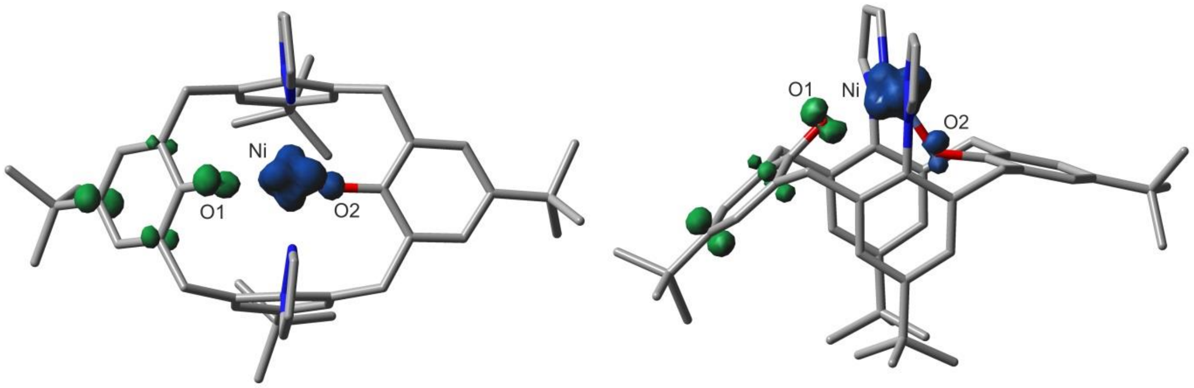

Figure S 21 – Optimised molecular structure and Mulliken spin density distribution of the asymmetric doublet state of [Ni(bpzCal)]$^{+}$. Top and side view. Hydrogen atoms are omitted for clarity.

Cartesian coordinates (Å) for the theoretical structure of of the unsymmetric triplet state (ground state) of [Cu(bpzCal)]$^{+}$:

B3LYP/Def2-TZVP, E = -3951.12821823 hartree

	x	y	z
Cu	0.583814	2.667050	0.013730
O	-1.603053	2.400964	0.001782
O	1.403210	0.874233	-0.001606
N	0.695208	3.183082	-1.895244
N	0.335795	2.297085	2.930160
N	0.686839	3.145255	1.931613
N	0.379794	2.341351	-2.910961
C	-3.142390	1.138127	-1.260058
C	-2.466712	1.471917	2.525441
C	-4.283558	0.384567	-1.230064
C	-4.919621	0.005999	-0.021924
C	-6.152340	-0.889911	-0.078374
C	-7.244834	-0.210218	-0.932305
C	-4.338883	0.421543	1.191213
C	-5.748206	-2.233727	-0.729083
C	-6.737065	-1.183157	1.310369
C	-3.188743	1.173693	1.235834
C	-2.578915	1.614013	-0.006876
C	-1.301901	0.498971	2.671881
C	-1.565340	-0.865520	2.656824
C	-0.557440	-1.828579	2.656439
C	-0.926962	-3.314949	2.712530
C	-2.007201	-3.634018	1.658993
C	0.283915	-4.224408	2.458710
C	-1.486004	-3.622447	4.117477
C	0.758463	-1.372929	2.618417
C	1.075132	-0.017647	2.653146
C	0.031952	0.906987	2.734405
C	0.370407	2.931545	4.122334
C	0.750417	4.237286	3.897094
C	0.936270	4.317816	2.513618
C	2.529077	0.400569	2.551055
C	3.191214	-0.008561	1.252584
C	4.387153	-0.687226	1.228124
C	5.044545	-1.024356	0.026626
C	6.364204	-1.793999	0.081517
C	6.940758	-2.078085	-1.312602
C	7.401432	-0.969770	0.875056
C	6.130882	-3.145180	0.792403
C	4.446570	-0.627982	-1.179899
C	3.245560	0.053264	-1.219274
C	2.569171	0.348348	0.009153
C	2.627754	0.505303	-2.525616
C	1.190652	0.046924	-2.679536
C	0.916398	-1.311610	-2.678364
C	-0.387135	-1.812879	-2.690270
C	-0.626950	-3.324759	-2.755720
C	-2.101794	-3.689407	-2.529500
C	-0.206355	-3.823915	-4.153633
C	0.220816	-4.041005	-1.685867
C	-1.424460	-0.887604	-2.647874
C	-1.200680	0.490007	-2.664397
C	0.114880	0.940198	-2.738117
C	0.414021	2.994137	-4.093046
C	0.755463	4.306129	-3.843213

C	0.920523	4.370641	-2.456256
C	-2.393155	1.430110	-2.535410
H	-3.153593	1.361643	3.366370
H	-2.105215	2.498315	2.517211
H	-4.703866	0.047013	-2.168560
H	-8.125691	-0.852587	-0.981328
H	-6.914186	-0.025178	-1.954295
H	-7.543912	0.743830	-0.495151
H	-4.790223	0.121087	2.125838
H	-4.965584	-2.727876	-0.150050
H	-5.382332	-2.102229	-1.747642
H	-6.612960	-2.898359	-0.769595
H	-6.030865	-1.721170	1.945638
H	-7.622488	-1.810837	1.205862
H	-7.040772	-0.269536	1.824509
H	-2.599232	-1.178836	2.628466
H	-2.218890	-4.704560	1.651516
H	-1.678864	-3.345017	0.661194
H	-2.946027	-3.118554	1.864075
H	1.047660	-4.106431	3.229028
H	0.743048	-4.028597	1.488388
H	-0.031898	-5.268342	2.472810
H	-0.740126	-3.417723	4.887573
H	-1.770046	-4.674433	4.189577
H	-2.368443	-3.018536	4.334827
H	1.577972	-2.074319	2.552776
H	0.129411	2.403452	5.028741
H	0.876124	5.016791	4.627225
H	1.232500	5.161027	1.912596
H	2.613226	1.483811	2.653233
H	3.089475	-0.034134	3.381333
H	4.839131	-0.967654	2.171110
H	7.159637	-1.158079	-1.857700
H	6.264583	-2.686549	-1.916246
H	7.876378	-2.629634	-1.213531
H	7.077572	-0.781007	1.898851
H	7.582991	-0.006606	0.394880
H	8.348527	-1.510634	0.921358
H	7.065609	-3.707150	0.838400
H	5.398215	-3.747938	0.252392
H	5.773072	-3.013249	1.813878
H	4.932965	-0.857247	-2.117359
H	3.227980	0.119147	-3.351776
H	2.683238	1.593751	-2.580719
H	1.756016	-1.993279	-2.646053
H	-2.218658	-4.773635	-2.550267
H	-2.745997	-3.279203	-3.309163
H	-2.464767	-3.334466	-1.563304
H	0.845443	-3.611709	-4.351008
H	-0.799102	-3.343693	-4.934382
H	-0.353086	-4.903368	-4.229665
H	1.289929	-3.930906	-1.867255
H	-0.001489	-5.109485	-1.683989
H	0.008781	-3.647042	-0.692561
H	-2.447474	-1.227280	-2.590747
H	0.199123	2.473695	-5.010377
H	0.869452	5.099798	-4.559886
H	1.185694	5.212366	-1.838860
H	-2.058258	2.465626	-2.531858
H	-3.060927	1.296820	-3.388645

Cartesian coordinates (Å) for the theoretical structure of of the symmetric triplet state of [Cu(bpzCal)]$^{+}$:

B3LYP/Def2-TZVP, E = -3951.12578267 hartree

	x	y	z
Cu	-0.000221	-2.749383	-0.001172
O	-1.999374	-2.317834	0.030239
O	1.999086	-2.317525	-0.032256
N	0.031157	-3.367749	1.906778
N	-0.074099	-2.501671	-2.943695
N	-0.031322	-3.365747	-1.909759
N	0.074118	-2.504714	2.941579
C	-3.150989	-0.674166	1.287982
C	-2.644846	-1.157331	-2.491231
C	-4.102965	0.316422	1.272720
C	-4.668279	0.814298	0.080678
C	-5.684318	1.952058	0.146825
C	-6.884730	1.522474	1.016953
C	-4.212783	0.270947	-1.129178
C	-5.001504	3.184825	0.782322
C	-6.211060	2.354447	-1.237701
C	-3.253288	-0.720277	-1.181189
C	-2.742353	-1.277872	0.043770
C	-1.321751	-0.422569	-2.671788
C	-1.311852	0.965653	-2.662087
C	-0.132398	1.710610	-2.678456
C	-0.199474	3.240479	-2.731362
C	-1.164477	3.763881	-1.648071
C	1.175262	3.889492	-2.513925
C	-0.725630	3.655950	-4.120948
C	1.067531	1.006215	-2.643097
C	1.115958	-0.387141	-2.675112
C	-0.091276	-1.084264	-2.747793
C	-0.094949	-3.165045	-4.120892
C	-0.063948	-4.514003	-3.837706
C	-0.024492	-4.585943	-2.439934
C	2.464238	-1.088490	-2.565739
C	3.150939	-0.672756	-1.288324
C	4.103001	0.317717	-1.272026
C	4.668416	0.814255	-0.079459
C	5.684548	1.951994	-0.144431
C	6.211386	2.352856	1.240499
C	6.884883	1.523233	-1.015074
C	5.001807	3.185496	-0.778587
C	4.212946	0.269628	1.129844
C	3.253342	-0.721529	1.180836
C	2.742247	-1.277729	-0.044720
C	2.644856	-1.159922	2.490404
C	1.321744	-0.425348	2.671604
C	1.311831	0.962875	2.662959
C	0.132366	1.707800	2.679786
C	0.199466	3.237640	2.733258
C	-1.175326	3.886731	2.516435
C	0.726075	3.652709	4.122781
C	1.164126	3.761276	1.649764
C	-1.067550	1.003424	2.643993
C	-1.115970	-0.389957	2.674945
C	0.091275	-1.087122	2.747042
C	0.095081	-3.169269	4.118105
C	0.063971	-4.517941	3.833567

C	0.024324	-4.588476	2.435729
C	-2.464258	-1.091208	2.564958
H	-3.320778	-0.914414	-3.312724
H	-2.488804	-2.233793	-2.490699
H	-4.417951	0.738819	2.218379
H	-7.610993	2.335305	1.073630
H	-6.585691	1.274803	2.035590
H	-7.382675	0.650509	0.589738
H	-4.604005	0.647854	-2.063384
H	-4.140407	3.501597	0.190341
H	-4.656954	2.983042	1.797001
H	-5.706281	4.016995	0.830307
H	-5.414020	2.724508	-1.885688
H	-6.941175	3.157289	-1.130168
H	-6.708293	1.522822	-1.740126
H	-2.262814	1.476655	-2.617932
H	-1.161882	4.855096	-1.638873
H	-0.869427	3.414511	-0.659179
H	-2.191484	3.441987	-1.822326
H	1.879808	3.620552	-3.302760
H	1.612277	3.607007	-1.554759
H	1.074404	4.975389	-2.526202
H	-0.057006	3.309902	-4.911207
H	-0.797736	4.743441	-4.187845
H	-1.716073	3.239988	-4.312044
H	2.004162	1.538797	-2.581195
H	-0.128797	-2.628969	-5.053545
H	-0.068817	-5.328663	-4.539816
H	0.008205	-5.452220	-1.800790
H	2.334085	-2.168329	-2.579557
H	3.086585	-0.816493	-3.420202
H	4.418010	0.741095	-2.217239
H	6.708575	1.520653	1.742009
H	5.414410	2.722290	1.888923
H	6.941563	3.155751	1.133789
H	6.585781	1.276684	-2.033964
H	7.382770	0.650766	-0.588816
H	7.611213	2.336061	-1.070907
H	5.706656	4.017654	-0.825708
H	4.140760	3.501712	-0.186238
H	4.657202	2.984828	-1.793468
H	4.604258	0.645492	2.064432
H	3.320746	-0.917836	3.312176
H	2.488811	-2.236384	2.488771
H	2.262779	1.473932	2.619154
H	-1.074475	4.972625	2.529104
H	-1.879665	3.617469	3.305345
H	-1.612592	3.604611	1.557275
H	1.716544	3.236626	4.313480
H	0.057662	3.306515	4.913154
H	0.798290	4.740177	4.189935
H	2.191225	3.439534	1.823768
H	1.161347	4.852491	1.640586
H	0.868906	3.411839	0.660943
H	-2.004181	1.536045	2.582468
H	0.129068	-2.634133	5.051293
H	0.068886	-5.333306	4.534858
H	-0.008515	-5.454110	1.795723
H	-2.334109	-2.171059	2.577696
H	-3.086559	-0.820055	3.419724

Cartesian coordinates (Å) for the theoretical structure of of the symmetric quartet state (ground state) of [Ni(bpzCal)]$^+$:

B3LYP/Def2-TZVP, E = -3818.95780084 hartree

	x	y	z
Ni	-0.001587	2.437576	0.037986
O	-1.872503	2.125102	0.041365
O	1.869814	2.127995	0.030408
N	0.008405	3.441203	-1.707945
N	-0.054301	2.510965	2.868527
N	-0.013043	3.370485	1.822691
N	0.051077	2.622711	-2.786189
C	-3.210228	0.676316	-1.249949
C	-2.648876	1.094888	2.510672
C	-4.201958	-0.280214	-1.256932
C	-4.766462	-0.794147	-0.073070
C	-5.831545	-1.887495	-0.160359
C	-7.031627	-1.376952	-0.985685
C	-4.282958	-0.300878	1.147170
C	-5.217590	-3.123545	-0.855571
C	-6.346273	-2.320266	1.219545
C	-3.286696	0.657120	1.211548
C	-2.751585	1.180697	0.001703
C	-1.297316	0.403952	2.648027
C	-1.275605	-0.983993	2.645494
C	-0.093909	-1.724049	2.647789
C	-0.150228	-3.253573	2.709002
C	-1.131989	-3.789835	1.647378
C	1.224665	-3.894667	2.469282
C	-0.648103	-3.663122	4.110973
C	1.100123	-1.012109	2.601917
C	1.145819	0.381252	2.626706
C	-0.067140	1.082322	2.687551
C	-0.072405	3.192924	4.034226
C	-0.042161	4.538318	3.733206
C	-0.005197	4.595352	2.334472
C	2.510522	1.053381	2.533428
C	3.207947	0.628833	1.261752
C	4.200037	-0.326830	1.230399
C	4.766816	-0.790515	0.027055
C	5.830879	-1.887524	0.070065
C	6.352567	-2.257454	-1.325384
C	7.027088	-1.417275	0.924292
C	5.211428	-3.153116	0.704866
C	4.285144	-0.246857	-1.172288
C	3.287484	0.711528	-1.198325
C	2.750110	1.183871	0.031404
C	2.648015	1.198913	-2.478884
C	1.297283	0.511291	-2.639588
C	1.277394	-0.876053	-2.677064
C	0.096844	-1.617392	-2.695454
C	0.157173	-3.145468	-2.778109
C	-1.218071	-3.792440	-2.557460
C	0.668781	-3.538400	-4.179520
C	1.130907	-3.688746	-1.712186
C	-1.098277	-0.909023	-2.632721
C	-1.146054	0.484371	-2.618425
C	0.065989	1.188615	-2.657325
C	0.068318	3.348881	-3.924755
C	0.036041	4.681696	-3.572105

C	-0.001194	4.684891	-2.172231
C	-2.511905	1.150905	-2.503259
H	-3.288269	0.818061	3.350445
H	-2.530251	2.176936	2.523170
H	-4.547607	-0.655429	-2.212031
H	-7.793523	-2.155532	-1.057328
H	-6.743221	-1.102137	-2.000562
H	-7.482464	-0.501725	-0.514894
H	-4.683163	-0.682719	2.075795
H	-4.360032	-3.499698	-0.293719
H	-4.883130	-2.897206	-1.868579
H	-5.958559	-3.922601	-0.921498
H	-5.550437	-2.740498	1.837878
H	-7.107035	-3.092430	1.099338
H	-6.803517	-1.489931	1.760687
H	-2.226239	-1.497515	2.621329
H	-1.128973	-4.881022	1.653529
H	-0.851293	-3.455482	0.649117
H	-2.156328	-3.465544	1.831983
H	1.941433	-3.620853	3.245260
H	1.643421	-3.611292	1.502101
H	1.129951	-4.981033	2.485162
H	0.032022	-3.306734	4.886709
H	-0.710215	-4.750618	4.186032
H	-1.638367	-3.253880	4.316913
H	2.040884	-1.537828	2.541202
H	-0.103652	2.670344	4.974687
H	-0.045772	5.361609	4.425108
H	0.026283	5.453578	1.684638
H	2.404151	2.136660	2.551707
H	3.112321	0.764582	3.396996
H	4.544690	-0.741073	2.169580
H	6.812000	-1.403311	-1.826098
H	5.560053	-2.649503	-1.966064
H	7.113016	-3.034148	-1.236435
H	6.733939	-1.189624	1.949413
H	7.481246	-0.521719	0.496937
H	7.787784	-2.199301	0.963556
H	5.951083	-3.955393	0.739015
H	4.356853	-3.502270	0.121457
H	4.870953	-2.972210	1.725004
H	4.686966	-0.589388	-2.115443
H	3.286526	0.955583	-3.329652
H	2.527611	2.280436	-2.449593
H	2.228458	-1.389122	-2.666577
H	-1.121327	-4.878308	-2.588006
H	-1.929760	-3.508552	-3.334518
H	-1.644407	-3.523366	-1.589456
H	1.658645	-3.122363	-4.373355
H	-0.006702	-3.177859	-4.957381
H	0.737117	-4.624769	-4.264829
H	2.157612	-3.368770	-1.891219
H	1.122660	-4.779896	-1.718892
H	0.847104	-3.352196	-0.715419
H	-2.038056	-1.437728	-2.585380
H	0.100433	2.862938	-4.884635
H	0.038510	5.530996	-4.231826
H	-0.034022	5.517480	-1.489925
H	-2.406993	2.234241	-2.479971
H	-3.111542	0.894411	-3.378481

References

[1] D. F. Evans, *J. Chem. Soc.* **1959**, 2003.

[2] V. Rawat, K. Press, I. Goldberg, A. Vigalok, *Org. Biomol. Chem.* **2015**, *13*, 11189.

[3] H. Hope, M. M. Olmstead, B. D. Murray, P. P. Power, *J. Am. Chem. Soc.* **1985**, *107*, 712.

[4] S. C. Goel, K. S. Kramer, M. Y. Chiang, W. E. Buhro, *Polyhedron* **1990**, *9*, 611.

[5] G. M. Sheldrick. SADABS; University of Göttingen, Germany, **1996**.

[6] G. M. Sheldrick, *Acta Cryst. A71* **2015**, 3.

[7] a) G. M. Sheldrick, *Acta Cryst. C71* **2015**, 3.; b) C. B. Hübschle, G. M. Sheldrick, B. Dittrich, *J. Appl. Cryst.* **2011**, *44*, 1281.

[8] A. L. Spek, *Acta Cryst. C71* **2015**, 9.

[9] M. J. Frisch, G. W. Trucks, H. B. Schlegel, G. E. Scuseria, M. A. Robb, J. R. Cheeseman, G. Scalmani, V. Barone, B. Mennucci, G. A. Petersson, H. Nakatsuji, M. Caricato, X. Li, H. P. Hratchian, A. F. Izmaylov, J. Bloino, G. Zheng, J. L. Sonnenberg, M. Hada, M. Ehara, K. Toyota, R. Fukuda, J. Hasegawa, M. Ishida, T. Nakajima, Y. Honda, O. Kitao, H. Nakai, T. Vreven, J. A. Montgomery Jr., J. E. Peralta, F, Ogliaro, M. Bearpark, J. J. Heyd, E. Brothers, K. N. Kudin, V. N. Staroverov, R. Kobayashi, J. Normand, K. Raghavachari, A. Rendell, J. C. Burant, S. S. Iyengar, J. Tomasi, M. Cossi, N. Rega, J. M. Millam, M. Klene, J. E. Knox, J. B. Cross, V. Bakken, C. Adamo, J. Jaramillo, R. Gomperts, R. E. Stratmann, O. Yazyev, A. J. Austin, R. Cammi, C. Pomelli, J. W. Ochterski, R. L. Martin, K. Morokuma, V. G. Zakrzewski, G. A. Voth, P. Salvador, J. J. Dannenberg, S. Dapprich, A. D. Daniels, Ö. Farkas, J. B. Foresman, J. V. Ortiz, J. Cioslowski, D. J. Fox *Gaussian 09*, Revision D.01, Gaussian, Inc., Wallingford CT, **2009**.

[10] a) A. D. Becke *Phys. Rev. A* **1988**, *38*, 3098; b) C. Lee, W. Yang, R. G. Parr *Phys. Rev. B* **1988**, *37*, 785; c) A. D. Becke *J. Chem. Phys.* **1993**, *98*, 5648.

[11] S. Grimme, S. Ehrlich, L. Goerigk *J. Comp. Chem*. **2011**, *32*, 1456.

[12] a) F. Weigend, R. Ahlrichs, *Phys. Chem. Chem. Phys.* **2005**, *7*, 3297; b) F. Weigend, *Phys. Chem. Chem. Phys.* **2006**, *8*, 1057.
